# Determination of Turning Radius and Lateral Acceleration of Vehicle by GNSS/INS Sensor

**DOI:** 10.3390/s22062298

**Published:** 2022-03-16

**Authors:** Juraj Jagelčák, Jozef Gnap, Ondrej Kuba, Jaroslav Frnda, Mariusz Kostrzewski

**Affiliations:** 1 Department of Road and Urban Transport, Faculty of Operation and Economics of Transport and Communications, University of Zilina, 010 26 Zilina, Slovakia; juraj.jagelcak@fpedas.uniza.sk (J.J.); kuba17@stud.uniza.sk (O.K.); 2 Department of Quantitative Methods and Economic Informatics, Faculty of Operation and Economics of Transport and Communications, University of Zilina, 010 26 Zilina, Slovakia; jaroslav.frnda@fpedas.uniza.sk; 3 Division for Construction and Operation of Means of Transport, Faculty of Transport, Warsaw University of Technology, St. Koszykowa 75, 00-662 Warsaw, Poland; mariusz.kostrzewski@pw.edu.pl

**Keywords:** turning radius, GNSS/INS sensors, vehicle, cargo, cargo securing, lateral acceleration

## Abstract

In this article, we address the determination of turning radius and lateral acceleration acting on a vehicle up to 3.5 t gross vehicle mass (GVM) and cargo in curves based on turning radius and speed. Global Navigation Satellite System with Inertial Navigation System (GNSS/INS) dual-antenna sensor is used to measure acceleration, speed, and vehicle position to determine the turning radius and determine the proper formula to calculate long average lateral acceleration acting on vehicle and cargo. The two methods for automatic selection of events were applied based on stable lateral acceleration value and on mean square error (MSE) of turning radiuses. The models of calculation of turning radius are valid for turning radius within 5–70 m for both methods of automatic selection of events with mean root mean square error (RMSE) 1.88 m and 1.32 m. The models of calculation of lateral acceleration are valid with mean RMSE of 0.022 g and 0.016 g for both methods of automatic selection of events. The results of the paper may be applied in the planning and implementation of packing and cargo securing procedures to calculate average lateral acceleration acting on vehicle and cargo based on turning radius and speed for vehicles up to 3.5 t GVM. The results can potentially be applied for the deployment of autonomous vehicles in solutions grouped under the term of Logistics 4.0.

## 1. Introduction

Accidents and damage to cargo occur due to the incorrect positioning or securing of cargo on vehicles. It is necessary to prevent cargo from sliding, tilting, or rotating in any direction during transport to protect human life as well as to protect cargo itself from damage. To identify the forces acting on a vehicle and its cargo, it is important to measure them using suitable devices such as accelerometers and gyroscopes. The aim of the paper is to study long average accelerations (minimum 1 s duration time) acting on a vehicle and cargo when cornering as the longest occurred lateral accelerations are only possible to be measured when cornering in long curves or in roundabouts.

This paper starts with a description of the current state-of-the-art connected to the determination of turning radius and lateral acceleration acting on vehicles of van type, together with cargo, in curves based on turning radius and speed. Further, a detailed literature review is given, including consideration of the research with application of the GNSS/INS (Global Navigation Satellite System with Inertial Navigation System). The first section is finalized with research gap specification. All the appropriate methods, measuring system, and preliminary evaluation of data are described in Section 2 in detail. The obtained quantitative results and appropriate equations based on the obtained measurements are presented in Section 3. The discussion is given in Section 4, and the paper is finalized with conclusions and future research presented in Section 5.

### 1.1. State-of-the-Art

The main research question of the article is to recognize whether it is possible to apply the available GNSS/INS sensor for monitoring the lateral accelerations acting on the cargo in road transport during cornering in curves and roundabouts and whether it is possible to calculate turning radius of vehicle up to 3.5 t gross vehicle mass (GVM) from Global Positioning System (GPS) coordinates by GNSS/INS sensor in order to calculate average lateral acceleration acting on a vehicle and cargo based on turning radius and speed.

There is no previous research for the determination of lateral acceleration of vehicle using procedures for evaluation of accelerations from the viewpoint of cargo securing. Therefore, in this paper, after conducting their own measurements, the authors establish the relationship between the turning radius determined based on acceleration and speed and the turning radius determined based on GPS coordinates, and based on this relationship, the authors propose a model to calculate the lateral acceleration for vehicles of GVM up to 3.5 t at a known turning radius and selected speed. The model is valid for turning radii of 5–70 m, with a radius of 23–27 m being the radius used for driving tests for the purpose of load securing according to EN 12642:2016 [1] and for stability testing of load units according to prEN17321:2020 [2].

The above-identified measurements’ explanation and research assumption led us to review and analyze the literature to define an appropriate in-fact research gap. We decided to search the most prominent scientific databases considering the following aspects. It was decided to scroll the literature with phrases such as “lateral acceleration(s)” coupled with “road vehicle(s)”, and additionally the main synonymous terms for linear infrastructure were included, namely: “roundabout(s)” or “curve(s)” or “traffic circle(s)”. Further, each of found numerous scientific contributions was deeply analyzed. The results of such analysis are explained below and summarized at the end of Section 1.2.

### 1.2. Literature Review

The safety of a heavy load vehicle, its driver, cargo, and equipment are significant during road transport processes. Nevertheless, as Alonso et al. [3] mentioned, the fleet-management systems are hardly fitted with objective and accurate measurements that would support avoidance of a particular accident. Instead of a constant threshold of speed or acceleration, the authors suggested that two mentioned indicators (or any other indicator) that cannot be exceeded to ensure safety are not enough. As the authors referenced to Biral et al. [4] typically tire–force saturation is the main combination of parameters that limits the dynamics and maneuverability of a particular vehicle. Maximum values of lateral and longitudinal accelerations combined with this set ensure to obtain an ellipse on a graph, where, e.g., maximum value of lateral acceleration is the semi-minor axis and maximum value of longitudinal acceleration is the semi-major axis of an ellipse. Consequently, in their research, Alonso et al. [3] suggested rather stability limits in the form of safe envelope boundaries consisting of rollover index, yaw rate and slip angle (safe envelope boundaries were applied in Alonso et al. [5], as well). Each of the parameters in this set has lower and upper limits, i.e., the range within which movement of the vehicle can be considered as safe. Boundaries were developed based on the speed of the vehicle, the steering-wheel angle, the yaw-rate error, and the sideslip angle error, and the tire–road friction coefficient. Boundaries characteristics changed collectively with friction coefficient.

Zhou et al. [6] presented a model of predictive control that takes into consideration road curvature information from a high-definition map and a vehicle speed preview control method which ensure speed decrease before a vehicle enters cornering road as, e.g., sine-shape roads, usually appearing in upland and mountains areas, and U-turn road, which can be treated as half of a roundabout. The authors have applied a performance index which includes typical (and critical) parameters, namely road curvature and yaw rate for road and vehicle state prediction, as well as lateral acceleration to control safety and ride comfort, and longitudinal acceleration typically applied for speed control (ride comfort with taking into consideration the mentioned parameters was also investigated by Xu et al. [7] for passenger vehicles). The authors assumed that their solution effects in automatic speed decrease before a vehicle enters a curve. This research was applied as a simulation method, and it considered especially passengers’ vehicles. Speed-limiting system based on lateral acceleration, combined lateral and longitudinal acceleration, and the vehicle’s performance capabilities were also given under simulation in Hamersma and Els [8].

To improve driver behavior and hence driving safety, Shu et al. [9] analyzed the effects of the features of horizontal curves on the steering behavior of passenger cars with a particular interest in free driving states: straight driving, variable curvature driving, and circular curve driving. This research was developed with the simulation method. What differentiated this research from several previously presented is the fact that the authors focused more on the geometry of road infrastructure in comparison to the dynamics of vehicles. Evaluation of driving style and its influence on fuel consumption was research by Loman et al. [10]. Jurecki and Stańczyk [11] analyzed longitudinal and lateral acceleration values to evaluate the driving style in the case of a passenger vehicle (a van). The authors developed their analyses in the actual domestic transport systems, including roads as urban areas (city), single roadways, expressways (with two roadways), and motorways. Note that the highest values and the greatest variety of values occurred for the urban area. The authors have not investigated straight and curved roads separately. Nevertheless, they resulted in certain acceleration thresholds. In contrast to previous research, the authors of Visar et al. [12] focused directly on curved roads. The authors considered lateral acceleration during rides of a passenger vehicle. Meanwhile, Xu et al. [13] analyzed distribution of lateral acceleration on three various types of roads, namely, a six-lane road, a four-lane road, and a two-lane road. Relations between lateral acceleration and curvature/radius were developed as well. This research was presented both for passenger cars, vans, and buses. Other researchers focus on passengers’ road vehicles of bigger sizes as coach buses. Tian et al. [14] focused on dynamics analysis of the critical conditions of rollover and sideslip of a large bus travelling on a curve. The effects of poor weather, wind, rain, and snow were analyzed with the application of a simulation tool. The general view of a vehicle as a multi-system was developed by Wallentowitz [15] and Rajamani et al. [16].

In addition, Sazgar et al. [17] considered typical dynamic changes of yaw rate velocity, longitudinal, and lateral accelerations. The authors focused on maneuverings, mainly on straight lines. They also applied their research as a simulation method, especially for passengers’ vehicles.

Ok et al. [18] recognized using sensors applied for positioning data or a six-dimensional inertial measurement unit (IMU) as originating too high cost intensity, therefore the authors developed a convolutional neural network (CNN) together with a dual-extended Kalman filter to estimate attitude of a vehicle. Their research were actual data applied in simulation modelling. Consequently, Zamfir et al. [19] analyzed dynamics of a vehicle riding on roundabouts and curves with use of different sets of IMU, and GPS applied in smartphones or Arduino equipment and compared it with professional equipment.

Maneuvering heavy-duty trucks in collision-free manner was a special interest of Shojaei et al. [20]. The authors presented their simulations of collision-free trajectories of a heavy-duty truck vehicle accelerating and surrounded by other vehicles, which were during either acceleration or deceleration. As in previously mentioned research, the dynamic model of the analyzed vehicle included the yaw rate, lateral velocity of the tractor, and articulation angle. Roundabout was not included in this research.

Whereas Shojaei et al. [20] considered a one-tractor one-trailer heavy-duty truck, Wang and He [21] focused on a multi-trailer heavy-duty vehicle (strictly writing, two trailers). The authors mentioned that ISO-14791 normative recommends the following maneuvers to be analyzed: an open-loop procedure with single sinewave steering input, and a closed-loop maneuver with a single sine-wave lateral acceleration input. Such maneuvers often lead to an asymmetric curve of tractor lateral acceleration coupled with a transient response; therefore, the authors suggested multiple cycles sinewave steering input. As in many previously presented cases, they applied simulation methods as well.

Anti-rollover system for heavy freight vehicle interest researchers for decades by realization of a low-cost solution for the rollover prevention in real heavy freight vehicles. Kamnik et al. [22] developed a digital simulator of the semitrailer which can be applied to enhance vehicle stability and road safety based on deeply investigated interpretations of signals given in the paper which formal modelling. Braghin et al. [23] investigated an anti-rollover control system involving parameters such as lateral acceleration and cross-wind force (cross-wind effect for passenger cars was analyzed e.g., in Lewington [24]). Such a system was generated to improve the stability of a heavy-duty vehicle which might be ensured by a vehicle-body roll angle reduction and the vertical load transfer. Simulation of anti-rollover strategy was presented as well. As in the case of Zhou et al. [6], the authors also investigated the U-turn road (which can be treated as half of the roundabout) under their simulation. When wind force is mentioned, it is also worth noticing the research of Balsom et al. [25] who considered the impact of wind forces on the stability of a heavy-duty truck—this time analyzes were developed based on actual measurements of speed, lateral acceleration, and roll angle of a vehicle driven around a highway loop-ramp. The authors mentioned that similar methods could be applied for studies of the impact of wind forces on truck rollover. The reaction of wind on a vehicle were investigated in detail by Zhang et al. [26]. The authors considered aerodynamic characteristics of crosswind forces on a heavy tractor semi-trailer under particular road conditions. Impacts of lateral acceleration, yaw rate, and the lateral displacement were analyzed with simulation methods. The lateral stability of a six-axle tractor-semitrailer was analyzed by Qu et al. [27]. A simulation model was compounded to confirm that a road’s radius and speed have essential effects on a vehicle’s lateral stability. Ikhsan et al. [28] also considered the dynamics of a heavy-duty vehicle, equipped with a wheelset consisting of from two to five axles. Using the simulation method, they found that a vehicle’s speed is characterized by a strong correlation to lateral acceleration which is one of the main reasons for a potential rollover. The willingness of developing an anti-rollover system has led Senalik and Medanic [29] to modify an existing semi-trailer air suspension system. The authors developed a promising simulation model. Ibrahim and Singh [30] considered minimization of rollover problem in liquid road tanker vehicles coupling dynamics of liquid and vehicle dynamics under different conditions such as braking and lateral acceleration (simulation research). Winkler et al. [31] studied field exploitation of six heavy-duty vehicles, five-axis semitrailers with tractors (cryogenic tanker for transport of liquid nitrogen); nevertheless, the authors focused on lateral acceleration measurements solely. Road tanker vehicles subjected the lateral acceleration were of interest by Romero et al. [32] as well. The authors investigated a device such as a hydraulic piston installed on a vehicle’s body to position the tilt table at different angles. Position and tilt angle were controlled through a valve in the hydraulic circuit. The authors applied the simulation method to investigate a vehicle that leaves a curved portion of the road and enters a straight segment.

Jiang et al. [33] presented a particular speed control system for heavy-duty vehicles which especially includes controls on upland and mountains areas with special care of sharp-curved roads. The authors presented a simulation-based assessment of the system which showed that this system ensures reduction of a peak temperature of brake drums, as well as a lateral slip angle of wheels, and a lateral load transfer rate of the vehicle body, which is strongly important especially in case of the unsymmetrically loaded vehicle (unevenly distributed load units on the vehicle). The system was analyzed so far for single frame heavy-duty vehicles; nevertheless, the authors plan to consider their system for more complex vehicles as semi-trailer trucks or tank trucks.

Cao et al. [34] focused on pitch dynamics and suspension tunings of a two-axle heavy vehicle with unconnected suspension. The authors considered a dynamic numerical model, especially to investigate front and rear suspension stiffness tunings with differentiated random road conditions and varied driving speeds, as well as occasionally braking inputs. Driving on curves, roundabouts have not been highlighted.

Tavassoli Kallebasti et al. [35] took attention to a vehicle skidding especially when a vehicle rides on a horizontal curve combined with vertical sag curves. The authors considered three types of vehicles: a sedan, a sports utility vehicle, and a truck, in simulation research. Since the maximum potential for skidding was reported for trucks, it is still worth analyzing.

Handling stability in tractor-trailers train to avoid tail flick, folding, lateral shimmy, or rollover was analyzed by Huang et al. [36] with the application of the optimization method.

Mischinger et al. [37] developed a method for analysis and assessment trajectory planning methods and controller types for usage in automated vehicles. These controllers occurred to be characterized by differences in lateral deviation and in the smoothness of lateral accelerations (as the authors mentioned, lateral accelerations have to be limited due to critical environmental conditions, e.g., a slippery road surface). The authors presented results obtained with the simulation method (which exchanged data between a path planner, a controller, and a vehicle model) and actual field test drives in the same roundabout as for simulations.

Nash et al. [38] focused on the literature review of driver control behavior modelling. As the authors mentioned, the aspects of sensory perception of the driver in the case of the driver–vehicle control had been neglected to date. Consequently, they presented a set of various sensory systems potentially applied to the mentioned modeling. Driver’s modeling for driving behavior analyses was reviewed previously by Plöchl and Edelmann [39]. Speed control modeling with application of driver’s model in simulation tool was investigated by Allen et al. [40], both for passenger cars and heavy-duty trucks.

In the sensing method employed by Eboli et al. [41], the speed and acceleration are fundamental parameters for estimating the driving safety combined with a driver’s behavior. Eboli et al. [41] proposed a methodology for the classification of a driver’s behavior based on the combination of speed, lateral and longitudinal acceleration (borderlines were developed). Common driver types were also analyzed with consideration of speed characteristics, handling load characteristics, and driving comfort on the road in Xu et al. [42].

The behavior of an autonomous vehicle, a vehicle with driver and a vehicle without driver (fixed steering wheel) were analyzed by della Rossa et al. [43]; however, this numerical-based research was considered by the authors with the application of the lateral stability solely.

Like rail transport, road transport should consider impacts due to the vehicle being moved, as well as external impacts, and ground impacts. The last one was numerically researched by Kavinmathi et al. [44]. The authors found that a curved road section sustains about two to six times more damage especially that during such maneuvering lateral load transfer is higher, and lateral forces increase as well. This also makes important to assess the actual center of gravity in a heavy-duty truck as Skrúcaný et al. [45] or Vu [46] presented it for a passenger car (with caring of other dynamic parameter for a particular vehicle), and Skrúcaný et al. [47] for a van (the authors investigated the freight position in the vehicle’s loading area, individual axles of such a vehicle, and braking deceleration). It is worth mentioning here that braking distance for various allocation of gravity center in case of real-world freight transport vehicle was considered by Skrúcaný et al. [48]. Changes of braking performance were also developed by Marienka et al. [49], and Ondruš and Kolla [50]. These topics, namely braking performance, center of gravity allocation, etc. are highly connected to freight transport safety during a vehicle ride—this challenge was analyzed by Vlkovský et al. [51]. As the authors mentioned, the insufficient cargo securing inside a vehicle resulted in one-fourth of all freight transport vehicle accidents in Europe. Such a topic was also investigated by Vlkovský et al. [52] for different types of roads. The authors of Vlkovský et al. [53] found that acceleration indicators given as normative ones can be exceeded even higher than it was predicted. Their analyses were applied with simulation methods.

Rouillard et al. [54] considered the approaches connected to the influence of shocks and vibrations on damage to cargo units in road transport. Consequently, based on the contribution of other researchers, they suggested changes to standards, norms, and test methods based on limited data obtained from specific transport scenarios. Taking into consideration the fact that vibrations generated by road transport vehicles are random, mainly due to the randomness of the road surface, root mean square parameter should be analyzed as it describes vibration severity. According to their opinion, the power density spectrum has rarely been considered in road transport, and they had not noticed formal attempt to compare the shape of a power density spectrum for vehicles and payload conditions. They suggested a root mean square and a power density spectrum to be included in a formal consideration.

The above-discussed literature references can be listed into following groups:

- Research focused on dynamics of passenger vehicles (including automobiles, vans, coach buses, etc.): Zhou et al. [6], Xu et al. [7], Hamersma and Els [8], Shu et al. [9], Jurecki and Stańczyk [11], Sazgar et al. [17], Visar et al. [12], Tian et al. [14], Ok et al. [18], Vu [46], Dižo et al. [55]—most of the mentioned research applied simulation methods, on the other hand, real conditions were included in Jurecki and Stańczyk [11], Xu et al. [13], Zamfir et al. [19].
- Research focused on dynamics of freight transport vehicles: Shojaei et al. [20], Wang and He [21], Braghin et al. [23], Lewington [24], Balson et al. [25], Zhang et al. [26], Qu et al. [27], Ikhsan et al. [28], Senalik and Medanic [29], Ibrahim and Singh [30], Winkler et al. [31], Romero et al. [32], Jiang et al. [33], Cao et al. [34], Tavassoli Kallebasti et al. [35], Huang et al. [36], Mischinger et al. [37], Marienka et al. [49]—most of the references listed before the hyphen applied simulation and numerical methods; however, results in field exploitation were presented, e.g., in Winkler et al. [31], Mischinger et al. [37], Skrúcaný et al. [48], Skrúcaný et al. [47], Vlkovský et al. [52], Vlkovský et al. [51].
- Research focused directly on drivers: Nash et al. [38], Plöchl and Edelmann [39], Allen et al. [40], Eboli et al. [41], Xu et al. [42], della Rossa et al. [43], Mikusova and Abnunazarov [56], Mikusova et al. [57], Kavinmathi et al. [44], Loman et al. [10], Rouillard et al. [54]—here it is worth underlining that research on drivers of freight transport vehicles was included solely in Allen et al. [40].

### 1.3. The State-of-the-Art for GNSS/INS

It is worth mentioning literature connected to the technology which is applied in the current research paper. The GNSS/INS is an answer to the challenges of GNSS signals attenuation, blocking or reflecting, as INS enables decreasing the pseudo-range gross errors [58]. As the authors of [59] mentioned, navigation with use a low-cost GNSS receiver and a microelectromechanical system-based inertial measurement unit (MEMS/IMU) is imminent for land vehicles, especially when navigation with satellites is insufficient. Consequently, GNSS/INS systems are characterized by extensive capabilities in application in between for road transport solutions as it was mentioned in [60]. Moreover, solutions based on GNSS/INS systems are constantly improved. The authors of [61] developed GINav, an open-source software which focuses on the data processing and analysis of a GNSS/INS integrated navigation system. The authors of [62] presented in-field road tests with two different MEMS inertial measurement units (IMU) to verify their strategy of excluding navigation errors. This strategy was applied as integration of the MEMS/INS, odometer and GNSS. The authors of [63] used a simplified algorithm of GNSS/INS for land vehicles applications, and the results conducted in their road test have shown that the degradation of the navigation accuracy caused by the mentioned simplification had a less impact on measurements than the sensors errors of the MEMS IMU. The major error sources in the case of GNSS/INS applications were analyzed by [64], what resulted in consideration of the initial attitude errors, accelerometer scale factors, gyro noise, and *g*-sensitivity errors (the results were given for short analysis period namely one second). Meanwhile, the authors of [65] developed adaptive Kalman filter navigation algorithm (RL-AKF), which has helped to reduce some of the errors. The authors indicated specific values for positioning errors in their article. The GNSS/INS was also validated with other solutions. For example, the main aim of [66] was to align all the available LiDAR trajectories of a road vehicle to confirm the of accuracy of GNSS/INS. The authors of [67] proposed innovative method to determine the railway track axis. Two GNSS receivers were applied what significantly improved precision of measurements and simplified the calculations of positioning corrections. Measuring wagon was moving at constant speed equaled to 10 km/h what enabled measuring the track inclination. In the case of mentioned research, for higher speeds further analyses are necessary. In [68], the authors stated that two antenna solutions are more relevant for position accuracy from the viewpoint of railway applications. These authors also researched difference between application of single-antenna and dual-antenna GNSS/INS devices in terms of vehicle speed. They concluded that dual-antenna GNSS/INS devices are less vulnerable to vehicle speed and can be applied in many railway applications.

### 1.4. Research Gap and Contribution of the Paper

The list of publications presented at the end of Section 1.2, which grouped the references [3,4,5,6,7,8,9,10,11,12,13,14,15,16,17,18,19,20,21,22,23,24,25,26,27,28,29,30,31,32,33,34,35,36,37,38,39,40,41,42,43,44,45,46,47,48,49,50,51,52,53,54,55,56,57,58], separates research on passengers from freight transport vehicles. It is worth mentioning that the dynamics of both types of vehicles are different and most of the screened research (not all of them are listed in this paper) consider passenger vehicles. This can be observed also in the case of driver-focus kind of research. Furthermore, most of these researches were based on simulation and numerical methods’ application. This paper focuses on light freight transport vehicles and is based on field exploitation results which is a strong contribution to the existing analyses. Moreover, it seems that so far, the topic of driving on roundabouts was neglected; in the found references only Mischinger et al. [37] considered such an infrastructure detail (driving on curves is more often applied). Based on this fact, the authors of this paper noted a research gap as deep analyses on the lateral acceleration acting on a freight transport vehicle and transported cargo when it was exploited on roundabouts and curves in the practical research field (the details are given in the methodology section of this paper).

## 2. Materials and Methods

The aim of this section is to present the application of GNSS/INS sensor for the calculation of turning radius from GPS coordinates calculated by GNSS/INS sensor (noted from now as R2) and calculation of turning radius from acceleration obtained from accelerometer of GNSS/INS sensor (noted from now as R1) and speed (obtained from GNSS/INS sensor). We also present the details of GNSS/INS sensor applied in the current research.

### 2.1. GNSS/INS Sensor Used to Measure Dynamic Actions

To measure dynamic actions when cornering with a vehicle, a dual-antenna industrial-grade dual GNSS/INS sensor VN-300 from company Vectornav was applied.

The VN-300 is a miniature, MEMS-based dual antenna GNSS-aided Inertial Navigation System that combines MEMS inertial sensors coupled with inertial measurement unit (IMU) which includes 3-axis gyroscope, accelerometer, magnetometer, and two high-sensitivity GNSS receivers. It combines acceleration and angular rates obtained from the IMU with position and velocity measured with use of GNSS receiver to provide optimal estimates of position, velocity, and orientation by a quaternion based proprietary Kalman filtering algorithms. By utilizing two separate GNSS receivers and antennas, the VN-300 enables accurate heading measurements without reliance on vehicle dynamics or magnetic sensors, providing unmatched performance under both static and dynamic conditions [69]. This sensor was also applied in [68]. Selected parameters of sensor are given in Table 1.

### 2.2. Forces to Design the Securing of Cargo

According to the standard EN 12195-1:2010 [70], the CTU Code [71] (CTU = cargo transport units) and directive VDI 2700 Sheet 16 [72], design lateral acceleration for road transport is 0.5 g for vehicles over 3.5 t GVM. Design lateral acceleration for vehicles up to 2.0 t GVM is 0.7 g and for vehicles between 2.0 and 3.5 t GVM is 0.6 g according to the directive VDI 2700 Sheet 16 [72]. For example, a lateral acceleration of 0.6 g means that it is necessary to secure 60% of the mass of the cargo in lateral direction.

The aim of the paper is to determine a calculation of lateral acceleration for passenger and cargo vehicles up to 3.5 t GVM based on turning radius and speed which means lateral acceleration up to 0.7 g.

### 2.3. Evaluation of the Measured Data

The sampling frequency of all parameters measured by GNSS/INS sensor was 200 Hz. Identification of different measured and calculated parameters used further in paper are given in Table 2.

### 2.3.1. Evaluation of Measured Lateral Acceleration

**Figure 1:**
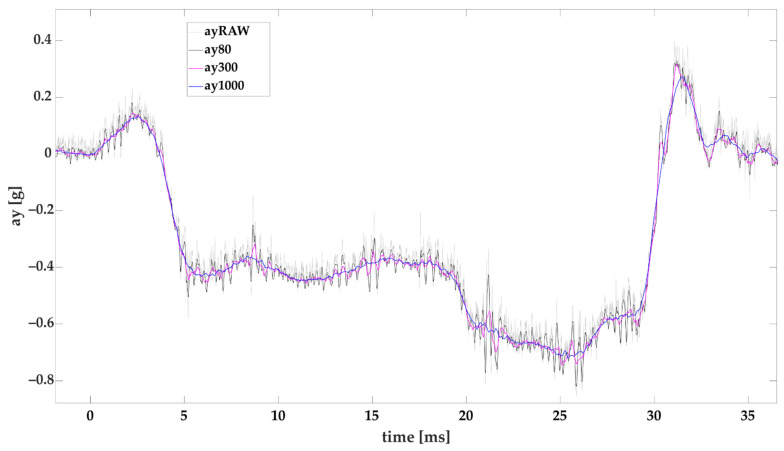
Effect of evaluation time on the lateral acceleration obtained from IMU during a vehicle’s cornering (ayRAW-raw data, ay80, ay300, ay1000—evaluation times 80, 300, and 1000 ms).

It is necessary to evaluate the raw data measured with sensor prior to their assessment and interpretation according to the standard EN 12195-1:2010 [70] which is used for road inspections of cargo securing in the European Union according to [73] as well as for the transport of dangerous goods according to [74]. Maximum/minimum average acceleration in 80 ms and 1000 ms are applied in relation to the EN 12642:2016 [1] standard, which is used in strength tests of superstructures or cargo securing by dynamic driving tests and 300 ms according to the standard prEN 17321:2020 [2] (examples are given in Figure 1).

#### 2.3.2. Evaluation of Turning Radius *R*1

The accelerometer data ay from IMU and GNSS/INS velocity data v are required to determine turning radius R1. The formula, given originally according to the work in [75], was modified for the purpose of the paper as follows:

(1) $$R1=\frac{v^{2}}{\left|ay1000\right|}\;[m]$$

The parameter ay1000 is used as absolute value for the calculation of R1 because lateral acceleration from the IMU has positive (in right curves) and negative values (in left curves). Calculated radius R1 is later averaged for each event determined by automatic selection of events.

The Equation (1) is basic equation for the calculation of lateral acceleration of vehicle not considering the inclination of the road and inclination of the vehicle. The vehicle inclines while turning due to the inertia forces and the positive or negative inclination of the road surface. The inclination of the road, inclination of the vehicle and the sensor height are considered in statistical evaluation of the results in Section 3 based on real tests. The inclination of the road surface is hardly possible to measure by the sensor used within the tests performed and solely total inclination of the vehicle together with road’s surface can be obtained. The inclination of the road and the inclination of the vehicle are hardly distinguishable.

#### 2.3.3. Evaluation of Turning Radius *R*2

**Figure 2:**
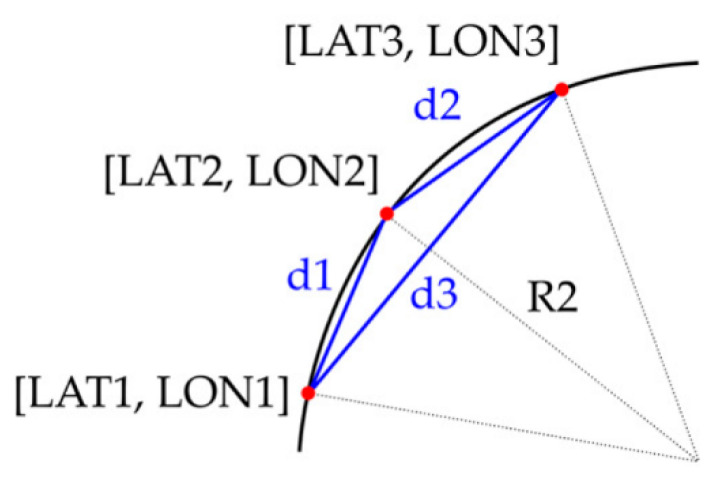
Turning radius R2 from circumscribed circle of a triangle defined by three points on curve.

GNSS/INS position data are required for calculation to determine turning radius R2. GPS coordinates of latitude (LAT) and longitude (LON) are necessary for calculation. Formula of curve radius computed based on three points (for circumscribed circle of triangle see Figure 2) was used to determine R2 as follows:

(2) $$R2=\frac{d1\cdot d2\cdot d3}{\sqrt{\left(d1+d2+d3\right)\cdot \left(-d1+d2+d3\right)\cdot \left(d1-d2+d3\right)\cdot \left(d1+d2-d3\right)}}\;[m]$$

where d1, d2, d3 are the distances in meters between three points of circular curve or the sides of triangle formed by this three points. The GPS coordinates of these three points are [LAT1,LON1], [LAT2,LON2], [LAT3,LON3] (Figure 2).

Haversine formula to calculate distances d1, d2, d3 of GPS coordinates was used as follows:

(3) $$d1=\cos^{-1}\left(\sin\left(LAT1\right)\cdot \sin\left(LAT2\right)+\cos\left(LAT1\right)\cdot \cos\left(LAT2\right)\cdot \cos\left(LON2-LON1\right)\right)\cdot 6,371,000\;[m]$$

(4) $$d2=\cos^{-1}\left(\sin\left(LAT2\right)\cdot \sin\left(LAT3\right)+\cos\left(LAT2\right)\cdot \cos\left(LAT3\right)\cdot \cos\left(LON3-LON2\right)\right)\cdot 6,371,000\;[m]$$

(5) $$d3=\cos^{-1}\left(\sin\left(LAT1\right)\cdot \sin\left(LAT3\right)+\cos\left(LAT1\right)\cdot \cos\left(LAT3\right)\cdot \cos\left(LON3-LON1\right)\right)\cdot 6,371,000\;[m]$$

where LATi is GPS latitude in radians and LONi is GPS longitude in radians. Calculated radius R2 is later averaged for each event determined by automatic selection of events.

Important part of the calculation is to decide what distance of points shall be used for calculation of R2. Based on own tests we decided to use floating window of the evaluation time of 2 s (400 samples) where [LAT1,LON1] is the first sample, [LAT2,LON2] is the 200th sample and [LAT3,LON3] is 399th sample. Fixed time window assures variable distances between points based on vehicle velocity.

#### 2.3.4. Automatic Selection of Events Based on Stable Lateral Accelerations (SEL1)

The aim of this selection is to determine events with a stable acceleration with a minimum duration of 1 s. For the purpose of selection, we use the following calculations:

(6) $$ay1_{k}=\underset{k}{\min}\left(ay1000i\right)\;[g]$$

(7) $$ay2_{k}=\frac{1}{k}\cdot \sum_{n-k+1}^{n}ay1000i\;[g]$$

(8) $$ay3_{k}=\underset{k}{\max}\left(ay1000i\right)\;[g]$$

(9) $$ay21_{k}=\left|ay2_{k}-ay1_{k}\right|\;[g]$$

(10) $$ay32_{k}=\left|ay3_{k}-ay2_{k}\right|\;[g]$$

where k is the window size of 200 samples which presents time window of one second, $ay1_{k}$ is the moving minimum of ay1000 for window k, $ay2_{k}$ is the simple moving mean-value of ay1000 for window k, $ay3_{k}$ is the moving maximum of ay1000 for window k, $ay21_{k}$ is the absolute value of difference between moving mean-value and moving minimum for window k, and $ay21_{k}$ is the absolute value of difference between moving maximum and moving mean-value for window k.

**Figure 3:**
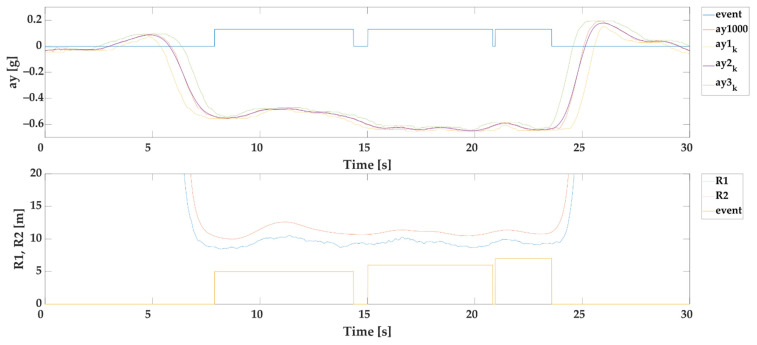
Example of automatic selection of events based on stable lateral accelerations.

Following conditions must be valid for selected events:

(11) $$\left|ay2_{k}\right|>0.1\;[g]$$

which means that average lateral accelerations of more than 0.1 g are evaluated and

(12) $$ay21_{k}\leq 0.035\;[g]$$

(13) $$ay32_{k}\leq 0.035\;[g]$$

which means that absolute value of difference between moving mean-value and moving minimum shall be less or equal to 0.035 g and absolute value of difference between moving maximum and moving mean-value shall be less or equal to 0.035 g over the window k. The value of 0.035 g was selected based on several different margins tested for all tested curves with radii from 5 to 70 m. The margin shall not be higher than 0.05 g according to the standard EN12642:2016 [1]. The example of SEL1 is given in Figure 3.

We decided to limit radiuses from 5 to 70 m because the highest accelerations occur for smaller radiuses during normal driving. Larger radiuses require substantially higher travelling speeds for equal lateral accelerations, which is not safe to test in normal road traffic. Larger radiuses errors do not have such significant impact on lateral acceleration as for smaller radiuses when considering Equation (1).

#### 2.3.5. Automatic Selection of Events Based on *MSE* of *R*1 and *R*2 (SEL2)

**Figure 4:**
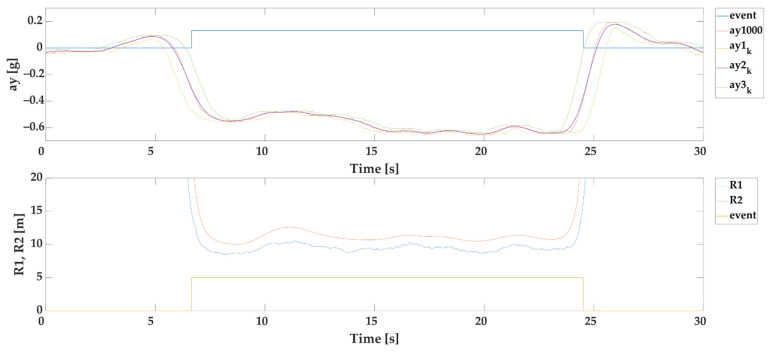
Example of automatic selection of events based on MSE of *R*1 and *R*2.

The aim of this selection SEL2 is to determine the events by calculating the MSE from the radii of rotation R1 and R2 with a minimum event duration of 1 s (example given in Figure 4). We calculate the MSE as follows:

(14) $$MSE=\frac{1}{n}\sum_{i=1}^{n}{\left(R1_{i}-R2_{i}\right)}^{2}\;[m]$$

Only events with MSE≤16 and minimum duration of event 1 s are further applied. We set the upper error boundary to 16 because it represents RMSE (Root Mean Square Error) as 4 m. Because the prediction range of proposed models starts from 5 m, we consider the 4 m error as a significant error (outlier) for further statistical modelling. MSE≤16 has shown as the most suitable for curves from 5 to 70 m.

Both selection of events require that minimum time of event shall be one second which means that with the increasing speed the minimum length of the curve increases, and shorter curves are not considered for evaluation.

### 2.4. The Methodology of Performed Measurements

The following part describes the vehicles and test routes used and description of tests performed during several days of testing.

#### 2.4.1. Test Vehicles

**Figure 5:**
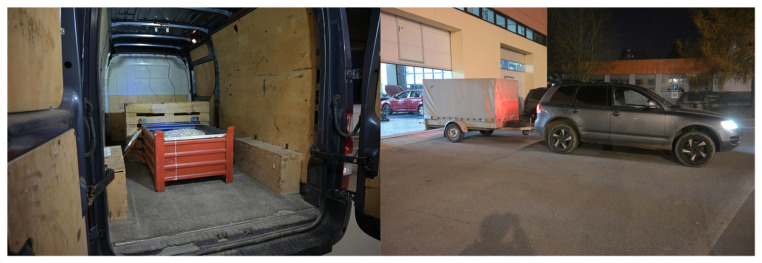
Van vehicle V7 (**left photo**) and vehicle combination V10 (**right photo**) used in tests.

Ten testing scenarios with eight different vehicles were applied for tests. The examples of field-test subjects (vehicles V7 and V10) are given in Figure 5. The sensor was installed in vehicle’s longitudinal axis on roof of passenger vehicles and in vehicle’s longitudinal axis under the roof of van vehicles with the antennas on roof. The highest lateral accelerations of the vehicle occur always when the sensor is allocated on the highest point in view of additional disturbances because of air-triggered movements and other external conditions. If the sensor is positioned lower than measured lateral accelerations are lower. The aim of the tests is to measure the highest possible results we can expect during regular driving.

**Figure 6:**
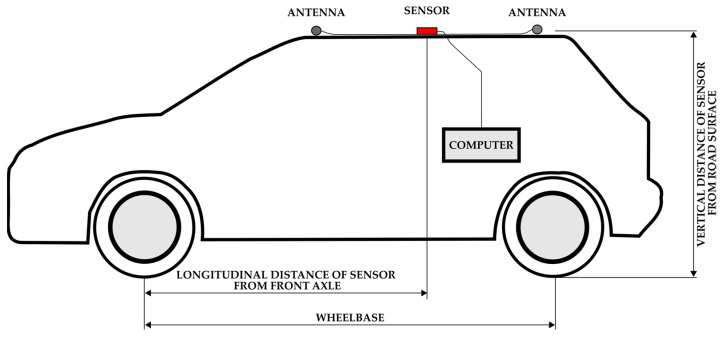
Scheme of test setup installation.

Van vehicles were tested loaded with 2 steel pallets of total mass 1000 kg (in Table 3 denoted as V5 and V7) and empty as well (V6, V8). Steel pallets with low center of gravity allowed to obtain the highest possible lateral accelerations without the vehicle tipping over. Vehicle V9 was tested without trailer and with 400 kg single axle trailer indicated as V10. Selected parameters of vehicles and position of sensor are indicated in Figure 6 and Table 3.

#### 2.4.2. Test Routes

**Figure 7:**
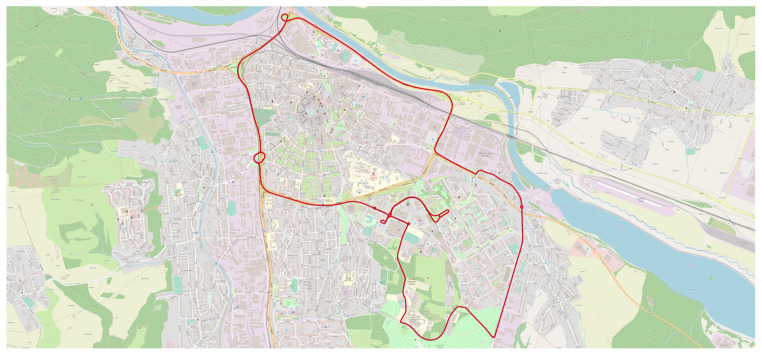
Test route TR1 in city of Žilina on OpenStreetMap (OSM) map layer.

**Figure 8:**
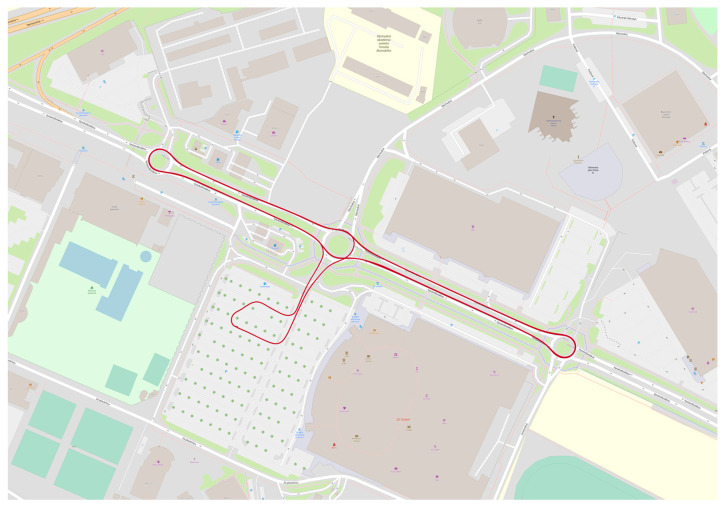
Test route TR2 in city of Žilina on street of Vysokoškolákov on OSM map layer with 4 passes of three roundabouts.

Two test routes were used in city of Žilina. Test route 1 (TR1; Figure 7) is a long route around the city of Žilina with the length around 17 km, meanwhile test route 2 (TR2) is a test route of three roundabouts on the street of Vysokoškolákov in Žilina with the length around 4.5 km. Each vehicle passed TR1 four times (except V3 with 6 passes) and certain vehicles passed also TR2 (one measurement is 4 passes; Figure 8). There are 42 test routes TR1 passed by 10 vehicles and 7 test routes TR2 passed by 7 vehicles. Total distance travelled by vehicles was 746 km. All tests were done during 6 nights of October and November of 2021 between 22:00 and 4:00 with the less possible traffic in city to pass the curves as fluently as possible.

Distances travelled by individual vehicles, measurements and test routes are given in Table 4.

TR1 was aimed at simulating real driving in urban conditions. A total of nine simple curves, two U-turns, five roundabouts, and one curve connector were driven on the route. The simple curves ranged in radius from 12 m to 65 m. The U-turns had radii of approximately 14.5 m and 16 m. A steady speed was maintained through all curves. The roundabouts had radii of approximately between 11 to 14 m and between 43 to 52 m. Two full turns were always made when passing through the roundabouts. The entry and first turn into the roundabout were at 25 km/h. For the second turn, the speed was increased to 30 km/h. When crossing a roundabout with a radius of 43 to 52 m, two turns were also made. However, the speeds were different, with the entry and first turn at 40 km/h and the second turn at 45 km/h. A steady speed was maintained during the roundabout crossing except in the area where the speed increase occurred. Driving through the car park area was also analyzed, where turns with small radii of around 12 m were observed at low speeds, which were not found anywhere else on our test route. TR2 focused only on roundabout crossings. A total of 4 passes through 3 roundabouts were made on the route. The first roundabout had a radius of 11 m and a U-turn was made, so a fourth exit was used. Two identical crossings were made on a 14 m radius roundabout, using the second exit. At the third roundabout with a radius of 12 m, the U-turn was used again.

#### 2.4.3. Statistical Investigation of Data

For statistical modeling, we needed to verify if there is a significant statistical difference between types of vehicles (V1 to V10). If not, we could use all data for modeling (based on R1 calculated values) without any concerns of biases in data. At first, the normality of data was assessed, which is a prerequisite for many statistical methods. For this reason, we applied the Kolmogorov–Smirnov test in Matlab (kstest). As a result, value equals to logical 1 was obtained, which means that the test rejects the null hypothesis (normal distribution) at the 5% significance level (*p*-value 0.05). This helped to choose the correct test for evaluation if samples originate from the same distribution. Because the data normality was rejected, a non-parametric test called Kruskal–Wallis test was used. Test computed the *p*-value to 0.295 (higher than 0.05), therefore it can be stated that type of vehicle (or better to say testing scenario) has no statistically significant impact on gathered data.

The MSE for model performance analysis was not the only one applied in the analyses. The statistical method *R*-squared (*R*^2^, or so-called the coefficient of determination) specifies to what extent variation of a predictable variable is described by the independent variable(s) in a regression model. *R*-squared coefficient is defined by formula below:

(15) $$R^{2}=1-\frac{RSS}{TSS}\;[—]$$

where RSS is the sum of squares of residuals (errors) and TSS is the total sum of squares.

To summarize, the following parameters are indicated in results of analyses:

- counts is the number of events from all vehicles and test routes;
- $R^{2}$ is the coefficient of determination;
- RMSE is the root mean square error;
- a is a slope parameter;
- b is the y-intercept;
- RES95 is the 95th percentile of absolute value of residuals (errors).

## 3. Results

In this section, we will focus on the interpretation of the measured and calculated results from parameters given in Table 2.

### 3.1. Turning Radius R*1* vs. R*2* of Events

**Figure 9:**
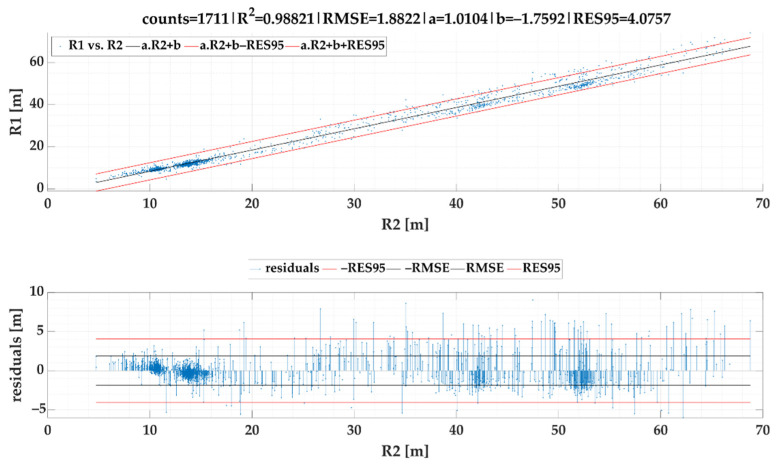
Turning radius R1 vs. R2 of events for *SEL*1.

**Figure 10:**
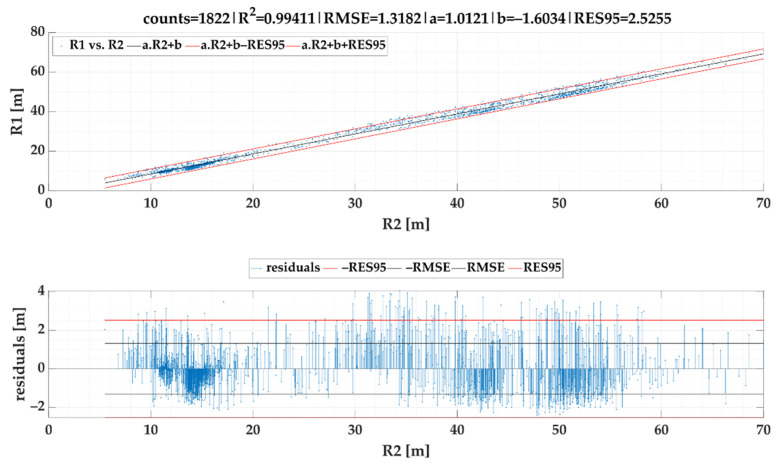
Turning radius R1 vs. R2 of events for SEL2.

The linear regression models of turning radiuses R1 vs. R2 are given in Figure 9 for SEL1 and Figure 10 for SEL2.

Figure 9 shows a linear correlation scatter plot between R1 and R2 as well as a residual plot for the best fitted linear regression model calculated from our dataset. The linear regression model produced an equation expressed as follows:

(16) $$R1c_{SEL1}=1.01037\cdot R2-1.75916\;[m]$$

The gained value of a coefficient of determination R2 was very high that interprets how well the proposed model fits the target (reference) data. RES95 verified good model veracity because 95% of all residuals lie below the absolute error of 4 m.

Based on the model given in Figure 9, the following formula for calculation of ayC can be stipulated:

(17) $$ayC_{SEL1}=\frac{v^{2}}{1.01037\cdot R2-1.75916}\;[g]$$

For *SEL*2 the linear regression model produced almost similar equation as *SEL*1 expressed as follows:

(18) $$R1c_{SEL2}=1.01211\cdot R2-1.60343\;[m]$$

Figure 10 shows results of the same modelling procedure, yet for the second selection. In this case, we received even more fitted results especially in a form of RMSE. *RES*95 is almost half as high as before. Based on the model given in Figure 10, the following formula for calculation of ayC can be stipulated:

(19) $$ayC_{SEL2}=\frac{v^{2}}{1.01211\cdot R2-1.60343}\;[g]$$

### 3.2. Calculated Lateral Acceleration ayC vs. Measured Lateral Acceleration ayM of Events

**Figure 11:**
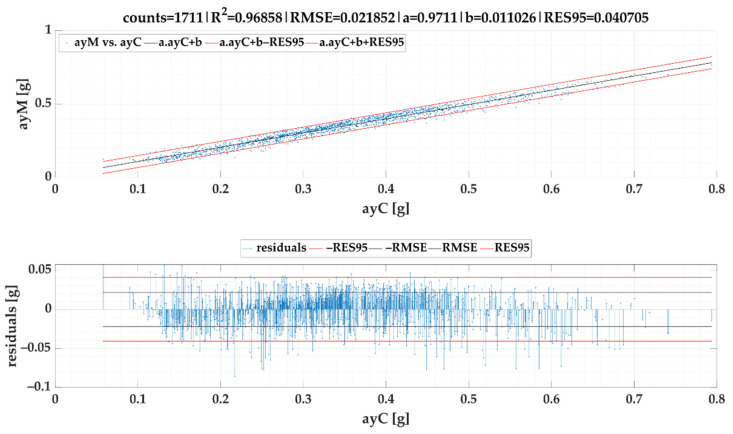
Calculated ayC vs. measured ayM of events for SEL1.

**Figure 12:**
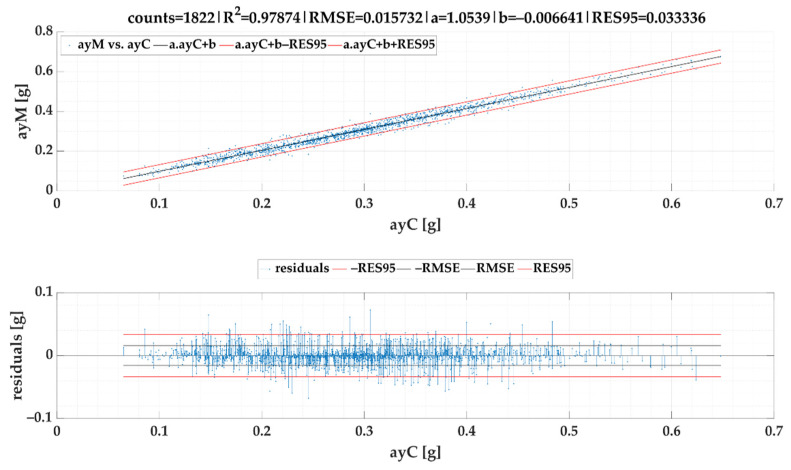
Calculated ayC vs. measured ayM of events for SEL2.

The linear regression models of lateral accelerations ayM vs. ayC are given in Figure 11 for *SEL*1 and Figure 12 for *SEL*2. Created models were included into the Equations (20) and (21) respectively. As, in the case of second selection (*SEL*2), longer steady-state periods are preferred, the lateral acceleration data range in *SEL*2 is lower. Both figures proved that regression models stated above provide high accuracy because RES95 in following calculations is only slightly higher than 0.04 g for *SEL*1 and 0.03 g for *SEL*2 when compared to a measured lateral acceleration *ayM*.

Based on the models given in Figure 9 and Figure 11, following formula for calculation of $ayMc_{SEL1}$ can be stipulated:

(20) $$ayMc_{SEL1}=0.97110\cdot \frac{v^{2}}{1.01037\cdot R2-1.75916}+0.01103\;[g]$$

Based on the models given in Figure 10 and Figure 12, the following formula for calculation of $ayMc_{SEL2}$ can be stipulated:

(21) $$ayMc_{SEL2}=1.05387\cdot \frac{v^{2}}{1.01211\cdot R2-1.60343}-0.00664\;[g]$$

### 3.3. Maximum Lateral Acceleration vs. Average Lateral Acceleration of Events

**Figure 13:**
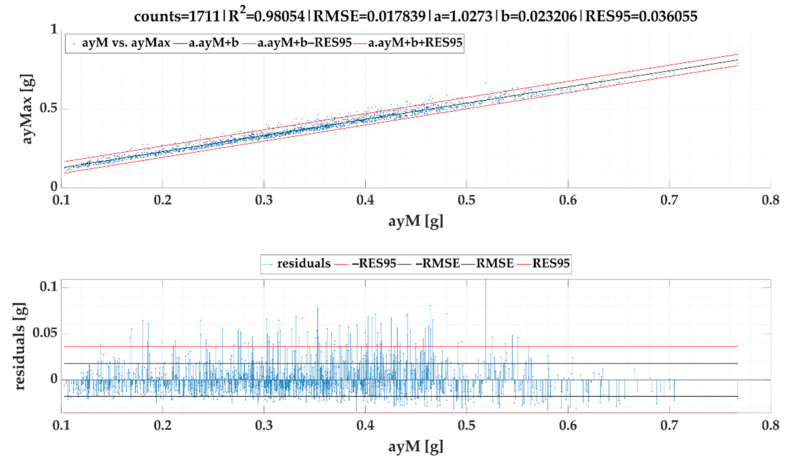
Maximum lateral acceleration ayMax vs. average lateral acceleration ayM of events for SEL1.

**Figure 14:**
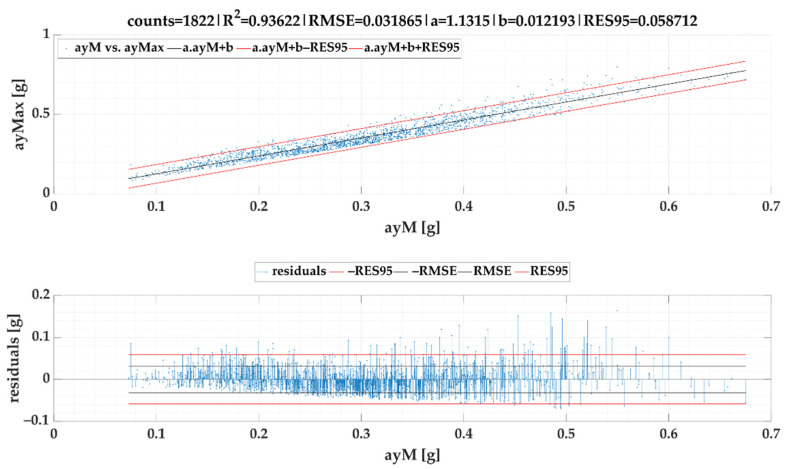
Maximum lateral acceleration ayMax vs. average lateral acceleration ayM of events for SEL2.

We also investigated the relation between maximum lateral acceleration and average lateral acceleration. As it can be observed in following figures, the linear correlation is very strong. The linear regression models of lateral accelerations ayMax vs. ayM are given in Figure 13 for *SEL*1 and Figure 14 for *SEL*2.

Based on the models given in Figure 9, Figure 11 and Figure 13, the following formula for calculation of $ayMaxc_{SEL1}$ were obtained:

(22) $$ayMaxc_{SEL1}=1.02734\cdot \left(0.97110\cdot \frac{v^{2}}{1.01037\cdot R2-1.75916}+0.01103\right)+0.02321\;[g]$$

(23) $$ayMaxc_{SEL1}=0.99765\cdot \frac{v^{2}}{1.01037\cdot R2-1.75916}+0.03453\;[g]$$

Based on the models given in in Figure 10, Figure 12 and Figure 14, we can stipulate following formula for calculation of $ayMaxc_{SEL2}$.

(24) $$ayMAXc_{SEL2}=1.13147\cdot \left(1.05387\frac{v^{2}}{1.01211\cdot R2-1.60343}-0.00664\right)+0.01219\;[g]$$

(25) $$ayMAXc_{SEL2}=1.19242\cdot \frac{v^{2}}{1.01211\cdot R2-1.60343}+0.00468\;[g]$$

## 4. Discussion

The tests were carried out to determine the long-term accelerations that can be achieved in normal road traffic driving, not driving tests according to EN 12642:16 [1] on test circuits. The longest accelerations in road traffic can only be simulated by cornering and in particular by multiple passages of roundabouts at different speeds. The aim of the tests was to perform night runs with available vehicles of M1, N1, and O1 categories on two test routes TR1 and TR2 and then to determine events by two types of automatic selection of events. Selection SEL1 is based on a steady acceleration in the interval +/− 0.035 g and selection *SEL*2 on MSE≤16 for radii R1 vs. R2. SEL1 is based on stable lateral acceleration to be more responsive to changes in speed and thus lateral acceleration on the second roundabout pass whereas *SEL*2 selects both roundabout passes as a single event and the resulting average values of lateral acceleration ayM, radii R1, and R2 are from both roundabout passes.

We are aware that performed tests have a few limitations. At first, there is not possible to pass the roundabouts exactly by the same path under real traffic conditions (this might be applied solely for numerical computation or simulation modelling). Second, according to the results depicted in Figure 9 and Figure 10, we collected solely a few data for radius ranging from 20 to 30 m. Field tests were conducted within one city that does not offer curvatures that belong to this range. On the other hand, a diagram of residuals pointed on balanced prediction performance within the whole model forecasting horizon.

It is worth comparing both selections of stable acceleration. Because of the second selection (*SEL*2) identified more stable acceleration periods, the regression model based on this selection offers a lower forecast horizon, yet it still meets directive VDI 2700 Sheet 16 [73] (lateral acceleration up to 0.6 g). More detailed look at models’ prediction accuracy discovers that *SEL*2 as a part of Equation (19) achieved a better correlation with the reference dataset than *SEL*1 used in Equation (17). Therefore, we recommend a regression model of lateral acceleration and turning radius determination based on *SEL*2 implementation.

If maximum event values ayMax vs. mean event values ayM is evaluated, in this case SEL1 achieves a lower RMSE and RES95 than *SEL*2, which results from the principle of event search by both selections.

The minimum and maximum measured lateral accelerations ay1000 for each vehicle in Table 5 are also important from the point of view of cargo securing (see Section 2.3.1). Data for ay80 and ay300 are also given.

All vehicles reach the highest values for ay80 and the lowest values for ay1000. M1-cathegory vehicles (V1, V2, V3, V4, V9) and empty N1-cathegory vehicles (V6, V8) achieved higher lateral acceleration values than loaded N1-cathegory vehicles (V5, V7) and the V10 vehicle with trailer. The highest lateral acceleration ay1000 was measured by vehicle V4 with value 0.798 g. Lateral accelerations from left curves are higher than from right curves for majority of vehicles. All minimum and maximum measured accelerations of individual vehicles are from test route TR1. All minimum lateral accelerations of tested vehicles were measured on roundabouts and U-turn.

## 5. Conclusions

In this paper, we aimed to identify dynamic events affecting vehicle and cargo for vehicles up to 3.5 t GVM from the point of cargo securing, which are also used for city logistics. The aim of the paper was to study long lateral accelerations when cornering and to find correlation model between turning radius from lateral acceleration and vehicle speed and turning radius from GPS coordinates. The measurements were carried out in the city, where there are frequent changes in the direction of travel, which affect the load and its packing. Based on this correlation model, we proposed the model of calculation of lateral acceleration of vehicle in curve from GPS coordinates of curve for given speed. The models of calculation of turning radius are valid for turning radius within range from 5 to 70 m for both methods of automatic selection of events with mean RMSE equals to 1.88 m for SEL1 and 1.32 m for SEL2. The models of calculation of lateral acceleration are valid with mean RMSE of 0.022 g for SEL1 and 0.016 g for SEL2.

To identify lateral acceleration forces acting on the vehicle and cargo, we selected GNSS/INS dual-antenna sensor to measure acceleration, speed, and vehicle position. This sensor was selected due to its appropriateness for research purpose, i.e., all parameters were recorded with a sampling frequency of 200 Hz and with repeatedly good and stable results of GPS coordinates.

Based on the presented findings, we can state that it is possible to use GNSS/INS devices not only to monitor cargo for its proper securing, but also to support the process of analyzing the condition of the road network based on turning radiuses to know expected lateral accelerations for given speed for vehicles up to 3.5 t GVM.

The main purpose is to use the results for the tests of cargo securing and transport stability of unit loads to know expected lateral accelerations in curves during normal driving. The results can also be used in monitoring of accelerations and turning radii for the purpose of cargo securing, e.g., when damages on cargo occur to know where the highest acceleration occurred and also to compare these accelerations with the accelerations affecting the transport stability of load units.

The results can also be used in the deployment of autonomous vehicles in city logistics, but also in manufacturing plants and logistics complexes. The used GNSS/INS sensor can be recommended for installation in autonomous vehicles for continuous data collection on the road infrastructure, which could increase the safety of freight transport and reduce the damage to the transported cargo. It can be defined that cargo is secured to defined acceleration, or transport stability of load units was tested to defined accelerations. Then autonomous vehicle shall operate in such condition that the defined accelerations are not exceeded. For example, current vehicles emergency braking systems do not take this into consideration, causing damage to cargo in such events. Here, also more conservative limits can be added to protect the cargo. There is also the advantage of autonomous vehicles because of the availability of the sensors for this purpose already installed in such vehicles. Modern vehicles have sufficient computing power to be able to calculate the radius of the curve and the lateral acceleration based on the radius and the data obtained from the sensors. The calculated values could then be compared with the accelerometer values and the vehicle would be able to learn from this comparison at what radius and at what velocity the given acceleration values would be reached. The longer the vehicle is in operation, the more data would be acquired and the more accurate the calculations would be.

The obtained results were promising; therefore, in future research, it will be possible to include heavier cargo vehicles into consideration, comparing the results based on the type of vehicle, and also the velocity of crossing through selected curves of the road network. For the sake of further research, it would be appropriate to compare the measured accelerations with varied positions of sensors in a vehicle which is specifically important for heavy vehicle combinations. It is not possible to achieve measured lateral accelerations over 0.5 g with heavy vehicle combinations during normal driving because very important point is the height of center of gravity where at lower lateral acceleration vehicle can tip over even at 0.25 g. Future studies should consider non-urban routes that provide curves and roundabouts with radii exceeding those considered in this article and also influence of vehicle and road inclination on lateral accelerations.

## Figures and Tables

**Table 1:** Basic functions and parameters of dual-antenna GNSS/INS sensor VN-300 from Vectornav [70].

Parameter	Value
GNSS-Compass Heading (1 m)	0.15–0.3°
Dynamic Heading	0.2°
Dynamic Pitch/Roll	0.03°
Gyro In-Run Bias (typical)	5–7°/h
Accel In-Run Bias	<0.04 mg
Accelerometer Range	±16 g
Gyroscope Range	±2000°/s
IMU Data	400 Hz
Navigation Data	400 Hz

**Table 2:** Measured and calculated parameters and sensors of GNSS/INS sensor VN-300.

Parameter	Parameter Identification [Unit]	Sensor/Calculation
Vehicle velocity	v [m·s^−1^]	output of GNSS/INS
Raw lateral acceleration of the sensor	ayRAW [g]	output of accelerometer of IMU
Average lateral acceleration during 80 ms	ay80 [g]	calculated output of accelerometer of IMU
Average lateral acceleration during 300 ms	ay300 [g]	calculated output of accelerometer of IMU
Average lateral acceleration during 1000 ms	ay1000 [g]	calculated output of accelerometer of IMU
Turning radius from vehicle speed and lateral acceleration	R1 [m]	calculation based on outputs of velocity from GNSS/INS and ay1000 of accelerometer of IMU
Turning radius from GPS coordinates	R2 [m]	calculation based on GPS coordinates from GNSS/INS
Calculated lateral acceleration of event	ayC [g]	calculation
Measured average lateral acceleration of event from ay1000	ayM [g]	accelerometer of IMU
Measured maximum lateral acceleration of event from ay1000	ayMax [g]	accelerometer of IMU

**Table 3:** Selected parameters of vehicles used in tests.

ID	Vehicle Name	Vehicle Category According to [76]	Manufacturing Year	Vehicle Mass [kg]	Wheel Base [mm]	Longitudinal Distance of Sensor from Front Axle [mm]	Ratio of Position of the Sensor and Wheel Base [mm]	Vertical Distance of Sensor from Road Surface [mm]
V1	VW Polo	M1	2006	1138	2441	1692	0.69	1480
V2	VW Polo	M1	2004	1033	2465	1721	0.70	1500
V3	Opel Antara	M1	2014	1941	2710	1815	0.67	1705
V4	Škoda Fabia	M1	2014	1116	2460	1780	0.72	1550
V5	Renault Master	N1	2019	3350	4325	3020	0.70	2320
V6	Renault Master	N1	2019	2350	4325	3020	0.70	2320
V7	Renault Master	N1	2014	3330	4360	2920	0.67	2355
V8	Renault Master	N1	2014	2330	4360	2920	0.67	2355
V9	VW Touareg	M1G	2003	2420	2865	1870	0.65	1713
V10	VW Touareg; trailer	M1G; O1	2003; 2005	2850	2865; 2818	1870	0.65	1713

**Table 4:** Distances in meters travelled according to vehicles, measurements, and test routes calculated from GPS coordinates.

Test Route	Measurement	V1	V2	V3	V4	V5	V6	V7	V8	V9	V10
1	1	16,864	17,251	16,908	17,331	16,999	16,687	16,944	16,924	16,697	17,031
1	2	16,582	16,948	16,905	16,925	16,959	16,939	16,935	16,923	17,030	17,037
1	3	16,966	16,964	16,926	16,932	16,976	16,967	16,945	16,933	17,054	17,035
1	4	16,966	16,962	17,375	17,354	17,265	17,252	16,949	16,936	17,024	17,029
1	5			17,445							
1	6			17,426							
2	1			4139	5048		4394	4509	4588	4588	4681
1&2	Total distance	67,380	68,127	107,127	73,594	68,204	72,244	72,289	72,313	72,402	72,823

**Table 5:** Minimum (left curves) and maximum (right curves) lateral accelerations ay80, *ay*300, *ay*1000 measured for individual tested vehicles.

Vehicle ay	Minimum Value [g]	Speed [km/h]	Maximum Value [g]	Speed [km/h]	Vehicle ay	Minimum Value [g]	Speed [km/h]	Maximum Value [g]	Speed [km/h]
**V1**					**V6**				
ay80	−0.855	32.1	0.724	43.4	ay80	−0.864	29.2	1.363	32.3
ay300	−0.700	27.9	0.685	45.0	ay300	−0.731	31.1	0.740	31.7
ay1000	−0.653	28.1	0.660	45.1	ay1000	−0.697	31.5	0.399	50.2
**V2**					**V7**				
ay80	−0.822	25.2	0.749	42.7	ay80	−0.682	23.3	0.670	30.0
ay300	−0.612	25.7	0.641	42.4	ay300	−0.606	24.7	0.493	39.7
ay1000	−0.596	25.5	0.563	42.7	ay1000	−0.526	24.3	0.473	39.7
**V3**					**V8**				
ay80	−0.857	24.6	0.820	35.2	ay80	−0.813	28.4	0.784	32.8
ay300	−0.655	49.1	0.532	47.5	ay300	−0.693	31.5	0.565	34.3
ay1000	−0.610	26.5	0.477	41.6	ay1000	−0.668	31.5	0.474	43.4
**V4**					**V9**				
ay80	−1.086	31.0	0.863	35.9	ay80	−0.829	27.2	0.545	30.7
ay300	−0.896	31.4	0.756	50.6	ay300	−0.775	27.9	0.433	41.2
ay1000	−0.798	30.9	0.720	51.1	ay1000	−0.746	27.9	0.401	41.2
**V5**					**V10**				
ay80	−0.913	29.3	1.061	26.1	ay80	−0.603	23.5	0.493	28.1
ay300	−0.706	28.7	0.651	26.5	ay300	−0.531	23.8	0.398	19.8
ay1000	−0.644	29.8	0.409	48.5	ay1000	−0.515	23.7	0.340	19.7
